# Iron Oxide Nanoparticles Coated with a Phosphorothioate Oligonucleotide and a Cationic Peptide: Exploring Four Different Ways of Surface Functionalization

**DOI:** 10.3390/nano5041588

**Published:** 2015-09-29

**Authors:** Frédéric Geinguenaud, Claire Banissi, Antoine F. Carpentier, Laurence Motte

**Affiliations:** 1 Université Paris 13, Sorbonne Paris Cité, UFR de Santé, Médecine et Biologie Humaine, F-93017 Bobigny, France; E-Mails: frederic.geinguenaud@univ-paris13.fr (F.G.); antoine.carpentier@aphp.fr (A.F.C.); 2 Université Paris Descartes, Laboratoire de Recherches Biochirurgicales, Hôpital Européen Georges Pompidou, F-75015 Paris, France; E-Mail: claire.banissi@aphp.fr

**Keywords:** iron oxide nanoparticles, peptide, oligonucleotide, surface functionalization

## Abstract

The superparamagnetic iron oxide nanoparticles (SPIONs) have great potential in therapeutic and diagnostic applications. Due to their superparamagnetic behavior, they are used clinically as a Magnetic Resonance Imaging (MRI) contrast agent. Iron oxide nanoparticles are also recognized todays as smart drug-delivery systems. However, to increase their specificity, it is essential to functionalize them with a molecule that effectively targets a specific area of the body. Among the molecules that can fulfill this role, peptides are excellent candidates. Oligonucleotides are recognized as potential drugs for various diseases but suffer from poor uptake and intracellular degradation. In this work, we explore four different strategies, based on the electrostatic interactions between the different partners, to functionalize the surface of SPIONs with a phosphorothioate oligonucleotide (ODN) and a cationic peptide labeled with a fluorophore. The internalization of the nanoparticles has been evaluated *in vitro* on RAW 264.7 cells. Among these strategies, the “«one-step assembly»”, *i.e.*, the direct complexation of oligonucleotides and peptides on iron oxide nanoparticles, provides the best way of coating for the internalization of the nanocomplexes.

## 1. Introduction

Since the discovery about 40 years ago that an antisense oligonucleotide could inhibit viral replication [1,2], only two synthetic phosphorothioates antisense oligonucleotides, Vitravene (1998) and Kynamro (2013), were approved by the Food and Drug Administration License. However, therapeutic efficiency of nucleic acids still remains unexplored due to several major drawbacks such as the specificity, the administration mode and side effects. This is mainly related to fast nuclease degradation and rapid elimination by the reticuloendothelial system (RES). To increase the metabolic oligonucleotide (ODN) stability, to reduce the RES clearance as well as to target specific tissues for therapeutic efficiency, many chemical modifications were explored such as substitution of phosphodiester functions by phosphorothioates moieties [3,4,5,6]. Still, large doses have to be injected, leading to side effects and potential toxicity. To prevent nucleic acid degradation [7,8,9], many vectors were also developed. However, except for viral vectors, most of these synthetic vectors were not found to be efficient for drug delivery [9].

Exciting opportunities are expected in using inorganic nanoparticles (NPs) in imaging (diagnostic) as well as gene and drug delivery. The NP surface is tailored to impart stability, biocompatibility and to crosslink other functionalities as fluorochromes (dual imaging), targeting ligands for cell-specific tropism and drug for theranostics application. Theranostics, the combination of therapy and diagnosis, is a new paradigm that emerged in nanomedicine a decade ago [10,11]. NPs originally designed for therapeutic purposes for the targeted drug delivery or medical imaging are now explored both as diagnostic nanotool (inherent to physical properties related to nanometer scale) and as treatment of the disease (nanocargo for the drug delivery) [12]. Among the variety of inorganic nanomaterials, gold and iron oxide NPs are especially studied for their potential as imaging contrast agent in optical imaging or computed tomography (CT scan) and magnetic resonance imaging (MRI), respectively [13]. Only iron oxide NPs are clinically approved.

For ODN delivery, silica, gold or iron oxide NP covered by a cationic polymeric and/or lipid layer are used [14,15,16,17,18,19,20,21,22,23,24,25], similarly to the strategy used for organic NPs. The cationic molecules allow both the ODN complexation via electrostatic interactions and the colloidal stability of the vector [14,15,18,21,26]. This approach, involving ionic interactions of relatively low energy, allows the ODN to be released passively into the cell. The most popular polymer used for the transport of nucleic acids remains the polyethyleneimine (PEI) considering commercial products [27,28]. However, it has also been revealed that polycations such as PEI or poly-lysine induce additional toxicity [29,30,31]. Finally, it has been shown that the addition of a cationic peptide, a cell-penetrating peptide, complexed at the surface of the NP via electrostatic interactions increased ODN penetration into the cells [27].

So far, the development of appropriate delivery systems still remains a challenge for DNA medicine.

**Scheme 1:**
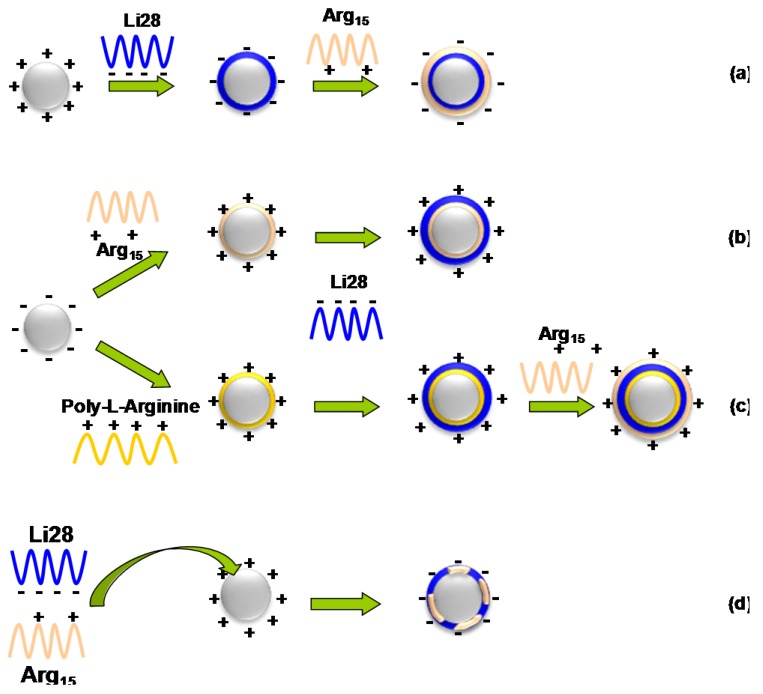
(**a**–**d**) four strategies used to functionalize iron oxide nanoparticle (NP) surface with a phosphorothioate ODN (Li28) and a cationic peptide (Arg_15_) via electrostatic interactions.

In a previous work, we develop a simple method allowing the direct complexation of a phosphodiester ODN on iron oxide NPs without the use of cationic polymers [32,33]. We have shown that depending on the ODN loading, two types of ODN stacking were observed: at low loading, the ODN interact with NP surface via phosphate groups and is fully adsorbed horizontally on the NP surface, keeping it hairpin structure but at higher loading corresponding to surface saturation (about 70 ODN per 10 nm in diameter NP) the ODN adopt a vertically ordered surface packing assembly, while maintaining double strand structure. In this work, we investigated whether these observations could be extended to a phosphorothioate ODN called Li28. To our knowledge, such a study was never reported in the literature. This ODN was already tested in a phase 1 trial for patients with recurrent glioblastoma [34]. Moreover, we explored various electrostatic interactions strategies in order to coat the NP surface together with Li28 and a cationic cell penetrating peptide. For the proof of concept, we used a peptide called Arg_15_ with 15 arginine residues. This choice was driven by a preclinical study that reported that the association of polyarginine with Li28 allows a better immune response [34]. Four strategies for Np surface functionalization were explored as summarized in Scheme 1. In a functional test, the intracellular internalization of the best candidates, from a physicochemical point of view, was evaluated *in vitro* on the RAW 264.7 murine macrophage cell line. Macrophages are the primary barrier for NPs, and the NPs efficiency internalization is a prerequisite for image tissue macrophages as cancer, Alzheimer, atherosclerosis, stroke, myocardial infarction, diabetes and other human diseases [35].

## 2. Results and Discussion

### 2.1. Nanoparticle Synthesis and Li28 Surface Complexation

To functionalize the surface of iron oxide NP with the phosphorothioate oligonucleotide (ODN) Li28, we used the electrostatic approach previously described [33], taking advantage of opposite charges carried by phosphorothioate groups (pK_a_ ≈ 1–2) and positively charge NP surface at pH = 2.5, Scheme 1a.

The nanoparticles with an average diameter of 10 nm are synthesized using the micelle route [36,37]. Their isoelectric point (IEP) is about 6–7. Below IEP, hydroxyl groups, coordinated to metal surface, are protonated inducing a net positive charge, +34 mV at pH = 2.5. This positive charge gives to the nanoparticles a good colloidal stability which results in a mean hydrodynamic diameter of 53 nm. To achieve the surface functionalization, the cationic nanoparticles dispersed in water at pH = 2.5 are mixed with the ODN dissolved in water at pH = 7 under stirring for 30 min. This process is performed with various amount of Li28, keeping a constant number of NP and defining the ratio *R* = Li28/NP. Under these conditions, the final pH is approximately 2.5.

Considering our surface functionalization process at pH = 2.5, we first check the impact of this treatment on the Li28 functionality. In fact, DNA is subject to depurination with a relatively high frequency under physiological conditions and hydrolysis of the N-glycosidic bonds is accelerated at low pH and high temperature [38]. Thus, acid hydrolysis has been reported at pH = 5 after heating at an elevated temperature (90 °C) for 6 h [38]. Phosphorothioate oligonucleotides, such as Li28, are known to be more resistant towards the enzymatic degradation than their phosphodiester counterparts. However, acidic hydrolysis of phosphorothioates has also been reported [39,40]. Moreover, the pK_a_ of adenine and cytosine are estimated equal to 3.8 and 4.5, respectively [41]. Hence, in our condition (pH = 2.5), these two bases are protonated, reducing hydrogen bonds with their complementary bases and, thus, this could promote the denaturation of the double helix structure.

#### 2.1.1. Li28 Structural Investigation at Acidic and Physiological pH

**Figure 1:**
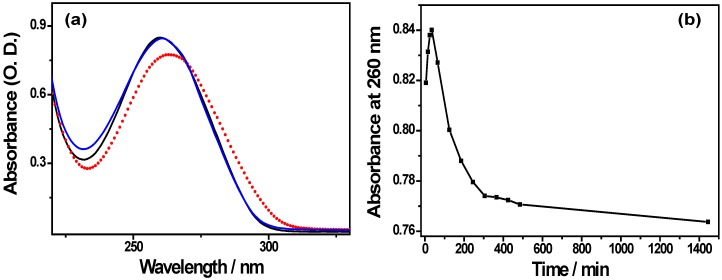
(**a**) UV spectra of Li28 at pH = 7.5 (black line), after 24 h at pH = 2.5 (red points) and 2 h after addition of NaOH for adjustment at pH = 7.5 (blue line); (**b**) Evolution of the Li28 absorbance at pH = 2.5 with time. [Li28] = 3 µM.

Li28 is characterized by an absorption peak at 260 nm due to heterocyclic purine and pyrimidine bases (Figure 1a). After solubilizing Li28 in water at pH = 2.5, the peak absorbance show time dependent variations as displayed Figure 1b. Three time dependent phases are observed (i) during the first 30 min, a slight increase of the absorbance; (ii) a decay phase until *t* = 5 h and (iii) finally a stabilization of the phenomenon. After 24 h, the UV spectrum shows a red shift in addition to absorbance decrease, (Figure 1a, red points). By adding a sodium hydroxide solution to go back to pH = 7.5, the UV spectrum of this solution recorded after *t* = 120 min is almost identical to the one obtained by solubilizing Li28 at pH = 7.5, (Figure 1a, black spectrum).

Various events can induce changes in the ODN absorption such as a structural conversion, denaturation of the double helix, oligonucleotide degradation as well as modification of the molar absorption coefficient induced by the protonation of nitrogen bases [41,42,43,44,45]. Thus, it has been reported a decrease of the nucleotide absorption involved in a double helix [41], compared to a single-stranded ODN and finally to a single nucleotide. In the case of irreversible degradation of the Li28 structure, an increase in absorbance due to cleavage of the DNA (release of free nucleotides) will be expected, or in the worst case a decrease in the absorbance associated with the destruction of heterocycles. However, the reversion to Li28 absorption at pH = 7.5 after 24 h in acidic medium suggest that Li28 is not degraded.

Taking into account these various parameters, the results presented in Figure 1b suggest that various incubation time events occur during the dissolution of the oligonucleotide Li28 in acidic medium. At short time (30 min), the increase in absorbance could reflect an opening of the double helix and then the decrease of the absorbance over a period of about 4–5 h could be attributed to the slow protonation of the bases. These results are consistent with those described by Zimmer *et al.* on DNA [44] and indicate that Li28 is not denatured.

**Figure 2:**
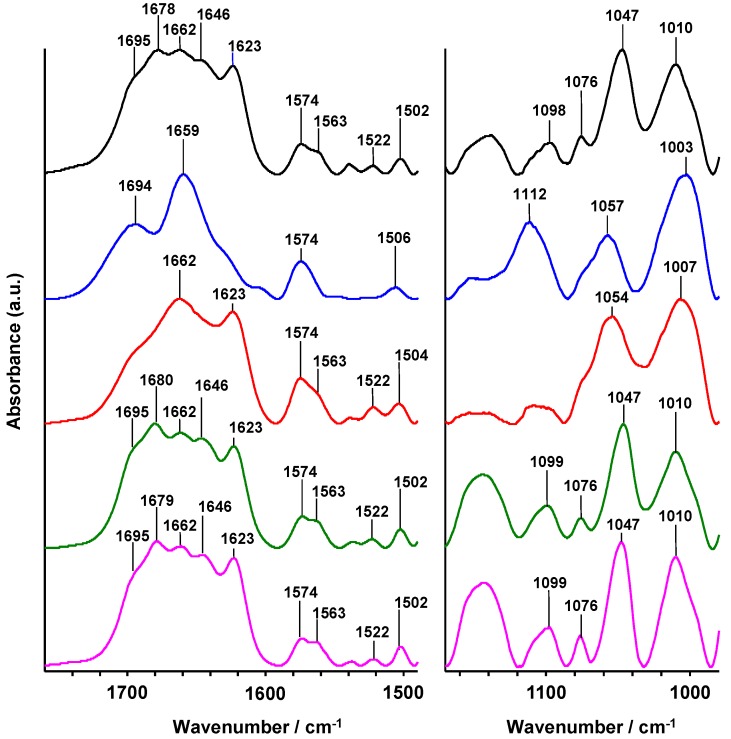
IR spectra of Li28 solubilized in D_2_O: at pH = 7.5 (black line); at pH = 2.5 (blue line); submitted to pH = 2.5 for 30 min before addition of NaOH to adjust the pH to 7.5 (red line); after acidic condition, readjustment at pH = 7.5 and then desalting process (green line); and after release from NP surface at a ratio *R* = 70 and desalting process (purple line).

To further investigate such assumption, the Li28 structure was studied using infrared (IR) spectroscopy. Figure 2 shows the characteristic IR vibration bands of base pairing (1500–1750 cm^−1^) and the conformation of the backbone (1000–1150 cm^−1^) recorded in D_2_O after incubation of Li28 in various pH conditions.

At pH = 7.5 (Figure 2, black spectrum), the spectrum is very close to that expected for a phosphodiester oligonucleotide backbone engaged in a double helix via a Watson-Crick base pairing. The characteristic bands involving at 1695 cm^−1^ the C2=O2 of thymine, 1678 cm^−1^ the C6=O6 of guanine, 1662 cm^−1^ the C4=O4 of thymine, 1646 cm^−1^ the C2=O2 of the cytosine and the thymine ring and at 1623 cm^−1^ the adenine ring are observed [46]. This result is not surprising because, except for substantial changes in the base stacking, related to the chemical modification influencing the hydration of the molecule, few changes are expected in the vibration frequencies reflecting the base pairing [47,48]. On the other hand, and in an expected way, drastic changes occur in the region 1000–1150 cm^−1^ sensitive to backbone conformation and characteristics of the vibrations of the phosphates and the sugars [46,49]. Considering this region, it may be assumed that the bands at 1047 and 1010 cm^−1^ are due to the C–O stretching vibrations of sugars, as observed in a phosphodiester backbone. To our knowledge, few data in the literature report infrared phosphorothioates signatures and vibration bands attribution is then rather difficult in this region. After incubation at pH = 2.5 for several hours (overnight), significant changes are observed in the IR spectrum (Figure 2, blue spectrum). In the spectral region from 1600 to 1700 cm^−1^, only two broad bands, multi-component, are present at 1694 and 1659 cm^−1^. According to Tsuboi and coworkers, IR spectrum of an ODN in acidic solution exhibits a shift of the adenine, guanine and cytosine vibration bands to larger wavenumbers [50]. Hence, at pH = 2.5, a strong vibration band of cytosine cycle is expected at 1658 cm^−1^ whereas the IR band of adenine ring is shifted from 1623 to 1668 cm^−1^. Thus, the IR spectrum in the bases’ vibration region reflects the expected ODN structural changes due to the acidic pH modification: protonation of the bases and denaturation of the double helix. Furthermore, considering the sugar-phosphate vibrations bands region, from 1000 to 1150 cm^−1^, the disappearance of the band at 1047 cm^−1^ concomitantly with the appearance of a band at 1057 cm^−1^ is observed. This latter band has already been observed at pH = 7 in a single-stranded ODN [47]. In such an event, shifting of the 1047 cm^−1^ vibration band toward a larger wavenumber could be considered, in the future, as an infrared marker for phosphorothioate ODN transition, from double helix structure to single-stranded ODN.

The change of pH solution from 2.5 (Figure 2, blue curve) to 7.5 by addition of NaOH (Figure 2, red curve) does not allow recovering the Li28 spectrum initially dispersed at pH = 7.5 (Figure 2, black curve). The spectrum obtained after adjustment at pH = 7.5 (Figure 2, red curve) is rather representative of a denatured oligonucleotide as supported by the observation of the band at 1054 cm^−1^. Such behavior could be due to salt screening limiting the re-formation of the double helix structure. This assumption is supported by the fact that after membrane ultracentrifugation in order to remove the excess of salt (NaCl) formed following the neutralization reaction, the spectra of Li28 (Figure 2, green curve) is very similar to initial IR spectrum (Figure 2, black curve). A slight difference is observed for C6=O6 guanines vibration band, observed at 1680 cm^−1^ after desalting compared to 1678 cm^−1^ in the initial conditions. This small difference could be due to the presence of Na^+^ ions in initial conditions. Indeed, the presence of Na^+^ ions, decreasing the repulsion between the negative charges carried by phosphorothioate groups, will contribute to the stability of the double helix structure by allowing a greater stacking.

Thus, in accordance with UV spectroscopy study, the IR spectroscopy analysis reveals no significant changes in the Li28 structure after acid treatment and we can conclude that the degradation of the oligonucleotide, following its incubation at pH 2.5 for 30 min, does not seem to occur. This assumption was also corroborated through agarose gel electrophoresis.

**Figure 3:**
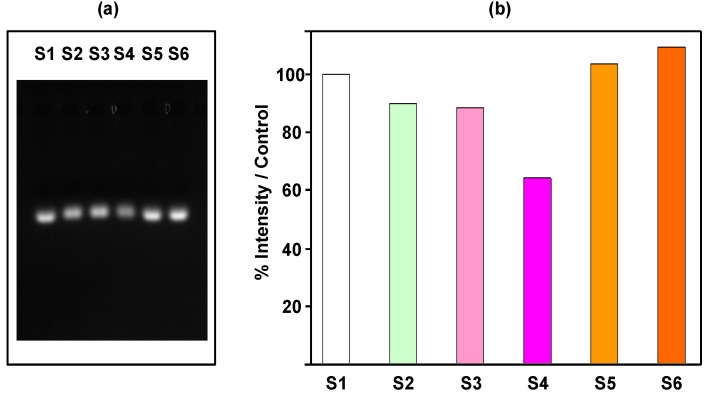
Agarose gel (1%) electrophoresis analysis of Li28 in various experimental conditions.

Electrophoresis is a well-known technique used to separate, under electric field, nucleic acids depending on their size and charge and related to their molecular weight. Thus, migration of a degraded nucleic acid is expected to be faster compared to its initial structure. Moreover, a single-stranded oligonucleotide will migrate in less time than a double helix structure. The migration of the Li28 was followed by fluorescence using the ethidium bromide (BET), a DNA intercalating dye [51,52,53,54,55]. Five different conditions (pH, salinity, *etc.*) were explored in order to study migration properties. In the control lane (S1), Li28 is solubilized at pH = 7.5, the second sample (S2) corresponds to Li28 dispersed in a 100 mM NaCl solution; for the third (S3) and fourth (S4) conditions, Li28 was incubated in a 0.1 M HCl solution for 1 min and about 4 h, respectively, and then neutralized with 0.1 M NaOH solution; the last sample (S5) corresponds to Li28 incubated for 30 min at pH 2.5, then the pH was rose to 7.5 and finally the solution desalted by membrane ultrafiltration (same sample as in infrared experiment, Figure 2, green curve). The results are displayed in Figure 3.

Comparing Li28 diluted in water at pH = 7.5 (S1) or in 0.1 M NaCl (S2), a 10% variation of the fluorescence intensity is observed. This result shows the sensitivity of the investigation method. When Li28 is dissolved at a pH of about 1 for one minute and then the solution neutralized with sodium hydroxide (S3), the fluorescence of the complex is found equal to Li28 dispersed with a similar salinity (S2). However, increasing acidic time incubation (S4) decreases the fluorescence intensity of about 25%. This indicates that under drastic pH conditions and time dependently, a significant deterioration of the ODN occurs. Finally, under the experimental conditions used for Li28 complexation on NP surface, the fluorescence of the complex Li28/BET after desalting process (S5) remains close to the control sample S1. In accordance with UV and IR investigations, this indicates that the Li28 double helix structure is maintained.

In conclusion, the fine structural study of Li28 in acidic medium shows that, in our experimental condition for electrostatic iron oxide surface complexation, the ODN double strand structure is maintained. Nevertheless, it should be emphasized that without any desalting procedure, the IR data will indicate ODN denaturation. This highlights the close and difficult relationship between chemical conditions and physical-chemical interpretations.

#### 2.1.2. Iron Oxide Surface Functionalization with Li28 ODN

The Li28 surface functionalization was performed at various molar ratio *R* = Li28/NP in order to evaluate the maximum NP loading. In a previous report, using a phosphodiester ODN in a hairpin structure and iron oxide NPs with an average diameter of 10 nm, the saturation of the surface was obtained for about 70 ODN/NP [33]. The increase of ODN density per NP up to 70 was correlated to a transition from the hairpin structure adsorbed horizontally on the NP surface to a vertically ordered surface packing assembly. We thus investigated whether such observation could be extended to phosphorothioate ODN. To our knowledge, such a study was never reported in the literature.

**Figure 4:**
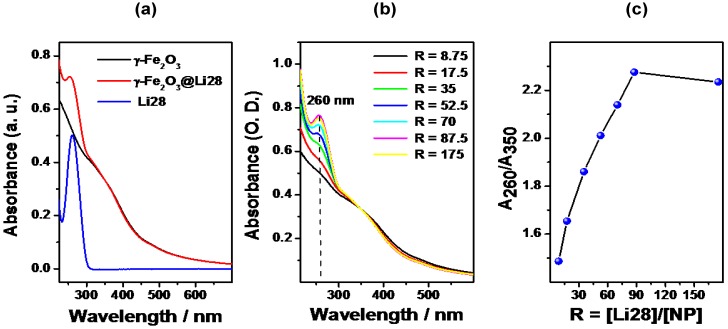
UV-Visible spectra of (**a**) uncoated NP at pH = 2, ODN and nanocomplex (R = 70) at pH = 7; (**b**) the nanocomplexes obtained for different values of R in water at pH 7 (spectra are normalized at 350 nm); (**c**) evolution of the ratio A_260_/A_350_ with ratio R.

Figure 4a shows the UV-Visible absorption spectra of the NPs and the Li28 before and after complexation (*R* = 70).

When dispersed in water (pH = 2.5), iron oxide NPs present a continuous absorption spectrum between 235 and 600 nm. The absorption spectrum of nanocomplexes (pH = 7) follows the additive property of absorbance, and a combination of the maghemite and oligonucleotide absorption is thus observed. Considering the spectrum of the oligonucleotide alone, no absorption is observed at 350 nm. Thus, the ratio between the absorbance at 260 nm (A_260_), representative of both ODN and maghemite, and that at 350 nm (A_350_), corresponding only to maghemite, should reflect the evolution of the ODN loading in function of the ratio *R*. An increase in A_260_/A_350_ ratio will be correlated with an increase in the number of ODN per NP.

The spectra of the nanocomplexes obtained with various *R*, dispersed in water at pH = 7.5, are presented in Figure 4b. An increase of the absorption at 260 nm with *R* is clearly observed. Figure 4c shows the evolution of the ratio A_260_/A_350_ *versus R*. The signal rose linearly with the increase of the molar ratio *R* up to 87.5 ODN strands per NP, whereby it reached a plateau. This indicates an increase of the number of ODN strands on the NP surface with ratio *R* and a saturation corresponding to an average of 87 ODN per NP.

The ODN loading was also evaluated by analysis of the UV spectra of the supernatants directly after the complexation process in acidic media (Figure S1 and Table S1). Below *R* = 87.5, almost 95% of ODN strands are adsorbed onto the NP surface.

The colloidal behavior, *i.e.*, hydrodynamic diameter and surface charge, of the nanocomplexes was determined by dynamic laser light scattering in water (pH 7.5) and the results are reported in Table 1. The average size decreases from *R* = 0 to *R* = 35 and then is stabilized at about 55 nm. Concurrently, a decrease of the ζ-potential (*Z*) is observed. The negative value (<−40 mV) indicates (i) the successful Li28 surface functionalization as the *Z* of uncoated NPs is close to zero at this pH; (ii) the stabilization of the NPs’ suspension due to electrostatic repulsive interactions and (iii) a better stability when the ODN density on the NP surface increases (ratio *R*).

**Table 1:** Values of hydrodynamic diameter (*Dh*), and ζ-potential (*Z*) measured in water (pH 7.5) at a 5 × 10^−4^ M iron concentration.

Values	*R*							
	0	8.75	17.5	35	52.5	70	87.5	175
***Dh* (nm)**	602	135	89	59	55	53	55	56
***Z* (mV)**	-	−42	−43	−45	−54	−54	−57	−48

Figure 2 and Figure 3 show the IR spectrum and electrophoresis migration, respectively, of Li28 after release from the NP surface and after desalting process (see experimental conditions). The IR spectrum (Figure 2, purple line) is very similar to the initial IR spectrum (Figure 2, black line), and the fluorescence of the complex Li28/BET (Sample S6 in Figure 3,) remains close to the control sample (Sample S1 in Figure 3,). These indicate that after NP surface complexation, Li28 keeps its double helix structure.

**Scheme 2:**
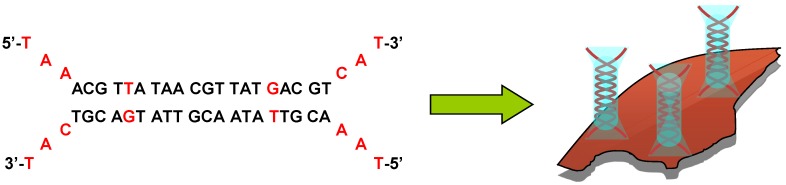
A possible conformation of the Li28 condensed at the surface of the NPs at high density in ODN.

Hence, our results show that, both with a phosphodiester [33] and a phosphorothioate ODN, it is possible to control the number of ODNs grafted to the surface of iron oxide NPs using an electrostatic approach. The maximum of loading was found equal to 70 for the phosphodiester ODN in a hairpin structure (intramolecular structure) and to 85 strands for the phosphorothioate Li28 ODN. Thus, considering the Li28 secondary structure, an intermolecular double helix, this indicates that the surface saturation of the NPs is obtained, on average, for only 42 double stranded structures. Concerning the orientation adopted by the Li28 at the surface of the NP, it is not possible to conclude, contrary to what it was done with the phosphodiester [33]. For such aim, it will be necessary to label the Li28 ODN with a fluorophore in order to study the fluorescence quenching evolution with ratio *R*. However, considering the evolution of the loading with R, as well as the double-stranded structure, a behavior similar to the one observed with the phosphodiester ODN (fully extended on NP surface at low ratio *R* and perpendicular organization with increasing *R*) could be expected. This hypothesis is supported by a geometrical model. In a duplex form, Li28 can be schematized as a cylinder with a radius of around 1 nm and a height of 8.9 nm [33,41]. The simplistic calculation, by considering the surface of the nanoparticle (314 nm^2^) and the base surface of the cylinder fully extended on NP surface (17.8 nm^2^), or in perpendicular configuration (3.14 nm^2^) shows that it is possible to coat roughly a maximum of 17 and 100 Li28 on the NP surface, respectively [33]. To explain the difference between phosphodiester and phosphorothioate ODN saturation, two parameters could be involved: the nature of the complexing group and the ODN structure. Considering the high complexation yield (95%), the results could not be related to lower interactions, compared to phosphodiester, of the phosphorothioate with iron oxide surface. Nevertheless, such an assumption needs to be confirmed through X-ray Photoelectron Spectroscopy (XPS) measurements, for example, in order to study surface coordination properties. The double stranded structure of Li28 is induced by the intermolecular association of 20 base pairs and six bases at the end are not paired. Thus, with our simplest geometrical model, the base surface area of the cylinder of the Li28 is much larger than that of the ODN phosphodiester used in the previous study, inducing a lower density per NP when the ODN is perpendicular to the surface (Scheme 2).

### 2.2. Li28 and Arg_15_ Peptide Complexation with Iron Oxide NP Surface

The ultimate goal of this project is to address our therapeutic nanovector toward specific areas of the body. For such an aim and in order to improve active targeting, peptides are commonly used [56,57,58,59,60]. As a proof of concept, we used a cationic peptide in order to generalize the electrostatic complexation process to both a phosphorothioate ODN and a peptide. This peptide, Arg_15_, consists of 15 arginine residues, and is labeled with a fluorescent TAMRA group. Due to the presence of the guanidinium groups (pK_a_ = 12.5) on the side chain, this peptide is characterized by a net positive charge at physiological pH. Arg_15_ is a cell-penetrating peptide that facilitates the cellular uptake of various cargos [61,62]. In this part, we will explore four different ways to functionalize the surface of NPs both with Li28 ODN and Arg_15_ peptide (Scheme 1). Considering that the iron oxide NP surfaces functionalized with Li28 ODN are characterized by a negative ζ-potential (Table 1), we first investigate the electrostatic interaction of the positively charged Arg_15_ peptide with negative Li28 nanocomplexes (Scheme 1a). This strategy was called as “step by step iron oxide surface functionalization”.

#### 2.2.1. “Step by Step” Iron Oxide Surface Functionalization with Li28 ODN and Arg_15_ Peptide

Scheme 1a presents our methodological approach. In a first step, NP surface was coated with Li28 corresponding to a ratio *R* = 60 using the conditions described above. In that case, it is expected that Li28 is perpendicular to NP surface. In the second step, the peptide is added to the nanocomplexes at pH = 7 corresponding to various amount of Arg_15_/NP: 0, 0.6, 6, 14 and 29 strands, respectively. The mixtures are stirred for 16 h, then centrifuged for 60 min and the supernatant is separated from nanocomplexes using a magnet. The analysis of the UV-Visible absorption spectra of the supernatants (Figure S2) shows a Li28 release on the order of 60% whatever the Arg_15_ amount used. The emission band characteristic of the TAMRA-labeled Arg_15_ peptide is localized at 575 nm (Figure S3). Using calibration curve for the fluorescent peptide alone (Figure S3) and measuring fluorescence intensity of supernatant (Figure S4), we can deduce the average number of peptides grafted per NP. A complexation yield around 80% is deduced (Table S2). Nevertheless, considering fluorescence intensity of the nanocomplexes, (Figure S5), it can be observed that high quenching occurs (>97%). For example, for the higher peptide surface complexation conditions (29 stands/NP), supernatant UV analysis indicates a complexation yield of 78% (about 23 peptides/NP) while the quenching is evaluated to be 99%. These results indicate, in our experimental conditions, a more efficient interaction of the peptide functions with the NP surface compared to phosphorothioate groups, which induced a ligand exchange process in which a part of the ODN strands are replaced by peptides. So, considering the greater interaction of Arg_15_ with the NP surface and the positive charge due to the guanidinium groups, we tried to first associate Arg_15_ to anionic NP surface, working at basic pH and then to complex Li28 ODN (Scheme 1b). However, even working at high Arg_15_ loading, the resulting nanocomplexes were not stable at physiological pH. This could be related to the low electrostatic repulsion interactions between NPs after the coating process. The excess positive charge provided by Arg_15_ coating on NP is not sufficient to provide good colloidal stability.

Due to the high extinction of the fluorescence signal and the low stability of the complexes, despite a high complexation yield of the peptide on NP surface, together with the high Li28 release, the so called “layer by layer assembly” was explored (Scheme 1c).

#### 2.2.2. “Layer by Layer” Iron Oxide Surface Functionalization with Poly-Arginine, Li28 ODN and Arg_15_ Peptide

Polycations are commonly used for nucleic acids cells’ transfection. The most commonly used is polyethylenimine. However, as explained in the introduction, a number of reports have indicated that PEI can induce cytotoxicity and could cause mitochondrial damages [29,30,31]. Recently, it was shown, using several polycations (poly-arginine, poly-lysine, poly-histidine and chitosan), that poly-arginine (PolyR) appears to be a good candidate for the formation of a ternary complex with Li28 and a short peptide called OVA made up of eight amino acids (SIINFEKL) [34]. In their work, authors showed that the complexation of the three components induces the formation of polymeric NPs with an average hydrodynamic diameter of about 400 nm. However, their formulation presents a time-dependent instability, as shown by the growing size of the particles, about 1 μm, which is observed after 10 hours of aging. Nevertheless, these particles demonstrated a greater stability compared to the Li28-OVA complexes and above all a better immune response, as well as a lower toxicity, compared to the other adjuvants. Compared to the Arg_15_ peptide used in this work, which is characterized by a net positive charge at physiological pH, the OVA peptide is characterized by a neutral net charge at pH 7. In order to overcome our first approach for the electrostatic assembly of both Li28 drug vector and Arg_15_ as a targeting agent onto the same one NP surface, we explore the so called “layer by layer” assembly. This approach, illustrated in Scheme 1c, consists of a three-step NP surface complexation. First, we try to functionalize the NP surface with PolyR using the anionic iron oxide properties’ surface at basic pH. At pH 10.5, the negative value of the ζ-potential of uncoated NPs, −52 mV, gives to the nanoparticles a good colloidal stability which results in a mean hydrodynamic diameter of 50 nm. Thus, the formation of cationic NP will be expected that could be used for further NP functionalization with anionic Li28 ODN and, finally, with cationic Arg_15_ peptide. We assumed, in a first assumption, that this strategy prevents, through high electrostatic interaction between PolyR and Li28 ODN, the release of Li28 during the Arg_15_ last step surface functionalization.

Briefly, for this “three-step surface complexation process”, first PolyR is added to negative NPs at pH 10.5. After incubation during 4 h at 37 °C under mixing, the non-complexed PolyR is removed using ultrafiltration. Bradford analysis of the supernatant, using a calibration curve (Figure S6), shows that complexation of PolyR with NP surface reaches a yield of about 80%, corresponding to an average of 140 PolyR per nanoparticle. At physiological pH, the average hydrodynamic diameter and zeta potential of γFe_2_O_3_@PolyR NPs are equal to 57 nm and +63 mV, respectively, indicating a successful complexation of PolyR with NP surface. Then, Li28 was added, corresponding to *R* = 17; 35; 69 at pH = 7 during 16 h at 25 °C under stirring (thermo mixing). In Table 2, we reported the hydrodynamic diameter and surface charge of the nanocomplexes.

The diameter increases and zeta potential decreases, when increasing the ratio *R*. These results reflect the efficiency of Li28 loading on NP surface and qualitatively indicate that the loading increases with the initial amount of Li28.

Finally, 9 Arg_15_ per NP were added to the previous nanocomplexes and the solutions were mixed for 3 h at 25 °C_._ Comparing colloidal behavior, no significant changes are observed (Table 2). This result has to be related to the excess of Li28 compared to the amount of Arg_15_ and to the weak sensitivity of scattering experiments.

**Table 2:** Values of hydrodynamic diameter (*Dh*), and ζ-potential (*Z*) measured in water (pH 7) at a 3.10^−3^ M iron concentration after adding PolyR, Li28 at different ratios and then Arg_15_.

*R*	0	17	17+Arg_15_	35	35+Arg_15_	69	69+Arg_15_
***Dh* (nm)**	57	58	57	60	59	69	68
***Z* (mV)**	+63	+59	+59	+52	+51	+50	+49

**Figure 5:**
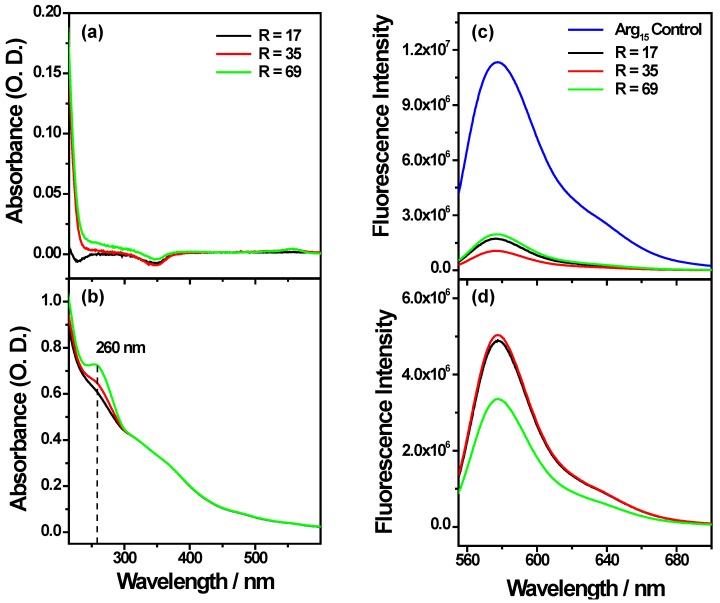
UV-visible (left) and fluorescence (right) spectra obtained for different values of *R* in water at pH 7 of (**a**,**c**) the supernatants; (**b**,**d**) the nanocomplexes (UV-visible spectra are normalized at 350 nm).

Figure 5 shows UV-visible and fluorescence spectra of the supernatants and nanocomplexes.

The characteristic absorption band at 260 nm of Li28 is not observed in supernatants and concurrently the nanocomplexes present an increased absorption at 260 nm with the ratio *R* (Figure 5). These indicate the efficiency of Li28 loading on NP surface without a release induced by Arg_15_ complexation. Finally, weak fluorescence intensity is recorded for the supernatants compared to the nanocomplexes, indicating a yield of coupling of about 85%–90% for Arg_15_. This indicates that about 7–8 Arg_15_ are grafted on the surface of one NP with a quenching effect (due to NP surface) of about 60%–70%, Figure 5c,d. This quenching effect is lower considering “layer by layer” assembly or direct Li28 surface functionalization. After incubation with 10% serum, no increase of fluorescence intensity is observed in our experimental conditions (Figure S7). This result seems to indicate that Arg_15_ release does not occurs due to further interaction. This assumption has to be related with previous results reported for the ternary system PolyR/Li28/OVA [34]. It was shown that only 4% of Li28 was released from the particles after three weeks of incubation.

Finally, in our investigation for Li28 ODN and Arg_15_ peptide association onto the NP surface, we tried to bind at the same time, on the NP surface, both the Li28 ODN and the Arg_15_ peptide (Scheme 1d).

#### 2.2.3. “One Step Assembly” Iron Oxide Surface Functionalization with Li28 ODN and Arg_15_ Peptide

For this strategy, we first solubilized Li28 with cationic Arg_15_ in water at pH = 7 with a molar ratio Li28/Arg_15_ of about 6.5. Thus, electrostatic interactions between anionic ODN and cationic peptide are expected. This solution was then added to NPs, solubilized in water at pH = 2 (Scheme 1d) under stirring during 30 min at 25 °C. In ours conditions, the theoretical composition of the ternary mixture is 58 Li28 and 9 Arg_15_ per NP. The hydrodynamic diameter and zeta potential of the γFe_2_O_3_@Li28_Arg_15_ NPs are 86 nm and −53 mV, respectively.

**Figure 6:**
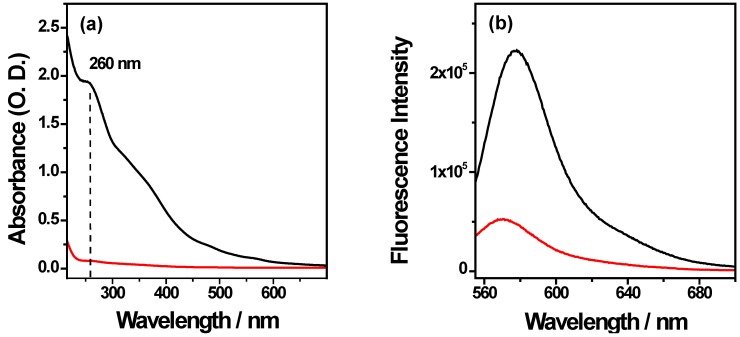
(**a**) UV-visible and (**b**) fluorescence spectra of the supernatants (red line) and the nanocomplexes (black line) in water at pH 7.

Figure 6 shows UV-Visible and fluorescence spectra of the supernatant and nanocomplex.

Clearly, UV visible spectroscopy analysis shows an efficient Li28 complexation on NP surface with a yield of about 98%. Considering our experimental conditions, a fluorescence intensity of about 1.2 × 10^7^ for the free Arg_15_ is expected. The emission band intensities measured in the supernatant and on the nanocomplex are about 5 × 10^4^ and 2.25 × 10^5^, respectively. These results indicate a high yield of complexation (99%) for the peptide on NP and after grafting a quenching factor of about 8.

**Figure 7:**
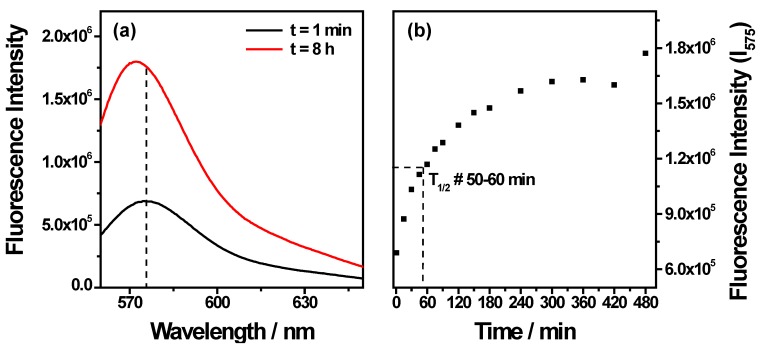
Fluorescence intensity of the nanocomplex in the presence of 10% serum.

Finally, Figure 7 shows the fluorescence spectra and time dependent fluorescence intensity after dispersion of the nanocomplexes in 10% serum. An increase of fluorescence intensity as a function of time is observed reaching a plateau at about 120 min. This indicates a release of Arg_15_ and a half time release at about 50–60 min.

### 2.3. Uptake of Nanoparticle by Macrophages

Considering the four strategies used to functionalize the NP surface with Li28 and Arg_15_, only the “layer by layer” strategy (γFe_2_O_3_@PolyR/Li28/Arg_15_) and the “one step assembly” (γFe_2_O_3_@Li28_Arg_15_) allowed obtaining nanoplatforms with good bio-stability and fluorescence properties. These two nanoplatforms exhibit a Li28/Arg_15_ ratio of about 6.5 and differ by their charge. Intracellular penetration of the NPs was assessed *in vitro*, on the murine macrophages, RAW 264.7 cell line. Cells were incubated with nanocomplexes γFe_2_O_3_@PolyR/Li28/Arg_15_ or γFe_2_O_3_@Li28_Arg_15_ ([Fe] = 175 µM corresponding to [NP] #9 nM) during 3 h followed or not by a 24-h wash-out period during which the cells were cultured in medium only. To monitor nanoparticle cell internalization, fluorescence microscopy and magnetic measurements were performed. Fluorescence microscopy allows imaging the Arg_15_ peptide labeled with TAMRA into the cells while the MIAtek reader (Magnetic Immuno-Assays Technology) is used to quantify the SPIO-NPs internalized in the cells [32,33,63,64,65].

**Figure 8:**
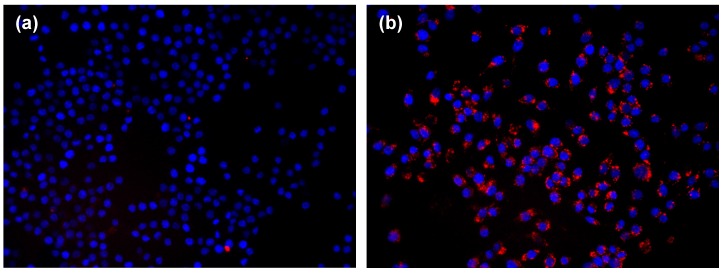
Fluorescence microscopy images of RAW 264.7 cells incubated with (**a**) γFe_2_O_3_@PolyR/Li28/Arg_15_ and (**b**) γFe_2_O_3_@Li28_Arg_15_ nanocomplexes. Nuclei are stained in blue with DAPI. Internalization of NPs, labeled with TAMRA, is seen in the cytoplasm (red dots). ×400 magnification.

With fluorescence microscopy, no evidence for internalization of TAMRA-labelled NPs were seen when γFe_2_O_3_@PolyR/Li28/Arg_15_ were used (Figure 8a). On the contrary, a clear cytoplasmic labeling was seen after a 3-h incubation with γFe_2_O_3_@Li28_Arg_15_, thus demonstrating that the NPs were internalized into the cells (Figure 8b). The labeling had almost completely vanished after a 24-h wash-out period (data not shown).

Similar results were obtained with the MIAtek technique which directly labeled iron NPs (Figure S8). The amount of iron internalized by the cells was deduced from a calibration curve (Figure S9). While minimal intracellular penetration was deduced with γFe_2_O_3_@PolyR/Li28/Arg_15_, γFe_2_O_3_@Li28_Arg_15_ NPs were easily detected (1.0 × 10^5^ and 1.5 × 10^6^ NPs/cell, corresponding to 0.1 pg and 2.8 pg, for γFe_2_O_3_@PolyR/Li28/Arg_15_ and γFe_2_O_3_@Li28_Arg_15_, respectively). Interestingly, the signal was still detected after the 24-h wash-out period (Figure S9); in contrast with the TAMRA labeling that was dramatically decreased in similar conditions. This discrepancy suggests that after penetration into the cells, the Arg_15_ peptide labeled with TAMRA is dissociated from the iron oxide NP surface, and is quickly metabolized.

The difference between both nanoplatforms might be correlated to charge surface and size effects. Indeed, phagocytic cells preferentially interact with negatively charged particles and display a greater uptake compared to positive particles [66]. Moreover, it has been shown that small superparamagnetic iron oxide particles are better ingested by macrophages compared to ultra-small superparamagnetic iron oxide particles [67]. The average size and charge surface of γFe_2_O_3_@PolyR/Li28/Arg_15_ and γFe_2_O_3_@Li28_Arg_15_ are 70 nm, +50 mV and 86 nm, −53 mV, respectively. Then, macrophages preferentially ingest the γFe_2_O_3_@Li28_Arg_15_ characterized by larger hydrodynamic size and negative surface charge.

## 3. Experimental Section

### 3.1. Physical Characterization

UV data were obtained using a Kontron Uvikon 942 spectrophotometer (Kontron, Paris, France). The extinction coefficients, expressed in L·mol^−1^·cm^−1^, used for the Li28 was 271,300 L·mol^−1^·cm^−1^ at 260 nm, and 420 for the iron at 480 nm.

Fluorescence measurements were performed using an excitation wavelength of 540 nm on a Spex FluoroMax spectrofluorometer equipped with a Hamamatsu 928 photomultiplier (HORIBA Jobin Yvon, Villeneuve D’Ascq, France).

Concerning the Fourier transform infrared (FTIR) spectroscopy, spectra were recorded on a Tensor 27 spectrophotometer (Bruker, Karlsruhe, Germany) at a resolution of 1 cm^−1^, and the data treatment was performed with the Opus program. Because of interfering vibrations of H_2_O at 1645 cm^−1^, spectra were recorded in D_2_O to study DNA base vibrations (1750–1500 cm^−1^). Deuteration experiments were performed by drying the samples and dissolving them in a D_2_O solution (>99.8% purity, Euriso-Top; CEA, Saclay, France). Solutions were deposited between two ZnSe windows. Samples were studied at an oligonucleotide concentration of 10 mM.

The mean particle size was determined by transmission electron microscopy. The size and the ζ-potential of the nanocomplexes were determined by dynamic laser light scattering on a Nano-ZS (Red Badge) ZEN 3600 device (Malvern Instruments, Malvern, UK).

For analyzing the potential degradation of the Li28 after complexation on the iron oxide nanoparticles, we have developed a process to release the ODN from the surface of the NPs. Firstly, the nanocomplex are incubated in 150 mM NaCl at pH 7 for three days at 25 °C. Then, the solution is centrifuged during 3 h and the supernatant is separated from the NP by magnetic separation. In this way, we have estimated that approximately 60% of the Li28 is released from the particles. Finally, the supernatant is desalted by ultracentrifugation using Amicon (Millipore) centrifugal devices with a molecular mass cut-off of 5 kDa.

For agarose gel electrophoresis, 30 µL of sample were mixed with 7.5 µL of 5× loading buffer containing 50% glycerol and 0.05% bromophenol blue (both reagents from Sigma-Aldrich, Saint-Quentin-Fallavier, France). 12.5 µL aliquots of the mixture (corresponding to 1 µg Li28) were then loaded in duplicate on a 1% agarose gel (Certified Molecular Biology Agarose) containing 0.5 µg/mL ethidium bromide. Electrophoresis was run for 60 min at 80 V in 1 TBE (45 mM Tris-borate, 1 mM EDTA, pH 8.0) running buffer (all electrophoresis reagents from Bio-Rad, Marnes-la-Coquette, France). The gel was then imaged using the ChemiDoc XRS+ Imager with Image Lab 2.0 Software (Bio-Rad, Marnes-la-Coquette, France).

### 3.2. Penetration of NPs into RAW264.7 Cells in Culture

RAW 264.7 cells were incubated *in vitro* in DMEM +10% FCS (both reagents from LONZA, Verviers, Belgium). Cells were incubated in the culture medium for 3 h with different NPs concentrations, followed or not by a 24-h wash-out period (in culture medium alone). Penetration of NPs into the cells was assessed at the end of the 3-h and the 24-h periods.

Intra-cellular TAMRA labeling was assessed by fluorescence. Briefly, 50,000 cells in 250 µL medium/well were seeded 8-well LAB-TEK I cell culture chambers (Thermo Scientific, Rochester, NY, USA). After the incubation periods, cells were washed three times for 5 min each with PBS, fixed for 5 min in 4% paraformaldehyde, then washed again with PBS. The nuclei were then stained for 3 min with 1 µg/mL DAPI (Invitrogen, Cergy Pontoise, France). TAMRA and DAPI fluorescence were visualized with Texas red and DAPI filters respectively, under a fluorescence microscope (Leica DM 2000, Leica Microsystems, Nanterre, France) using a 400× magnification.

Intra-cellular penetration of NPs was also assessed using Miatek reader (Magnetic Immunoassays Technology) [32,33,66,67]. Briefly, 250,000 cells in 1250 µL medium/well were seeded on 24-well cell culture plates (TPP, Trasadingen, Switzerland). After the incubation periods, cells were washed with PBS, and harvested by gentle dislodgement with a fine tip plastic Pasteur pipette. The Miatek sensor measures a signal that is proportional to the third derivative of magnetization at a zero magnetic field. Biological samples exhibit virtually no magnetic background; consequently, the MIAtek signal is directly correlated with the amount of magnetic particles, using a calibration curve established with the SPIO-NP stock solution at various concentrations (Figure S9). The quantity of iron internalized by the cells was deduced from the MIAtek calibration curve.

## 4. Conclusions

In this study, various methodologies, based on electrostatic interactions, are explored in order to functionalize the surface of iron oxide nanoparticles, with a phosphorothioate oligonucleotide Li28 and a cationic peptide Arg_15_ labeled with a fluorophore. Up to 42 double stranded Li28 structures are adsorbed onto the 10 nm particle surface. Considering a perpendicular surface packing assembly, this lower loading saturation, compared to previous results obtained with a phosphodiester ODN [33], was related to larger Li28 base surface area due to unpaired bases. Two nanoplatforms with a Li28/Arg_15_ ratio of about 6.5 presenting good bio-stability and fluorescence properties and differing by their charge were elaborated. *In vitro* evaluation of Murine macrophage cells shows a preferential uptake of the nanoplatform with a negatively charged surface. This work paves the way for the elaboration of a targeted and therapeutic bimodal imaging agent.

## Supplementary Materials

Supplementary materials can be accessed at: http://www.mdpi.com/2079-4991/5/4/1588/s1.
