# Supplementary material for: Iron Oxide Nanoparticles Coated with a Phosphorothioate Oligonucleotide and a Cationic Peptide: Exploring Four Different Ways of Surface Functionalization
Source: Nanomaterials (Basel). 2015 Sep 29;5(4):1588–609. doi: 10.3390/nano5041588 (PMC5304778; doi:10.3390/nano5041588)
Supplement: Supplementary file 1 [file nanomaterials-05-01588-s001.pdf]

Supporting Information

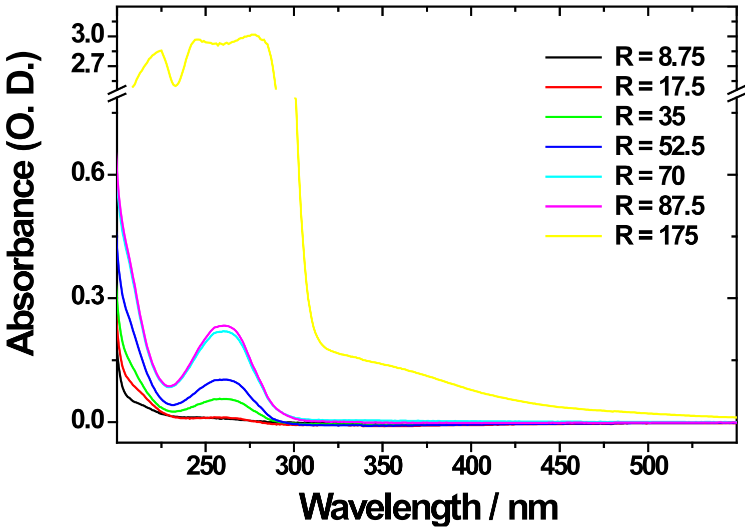

Figure S1. UV-visible spectra of the supernatants after complexation of Li28 with iron oxide nanoparticles for the different values of *R*.

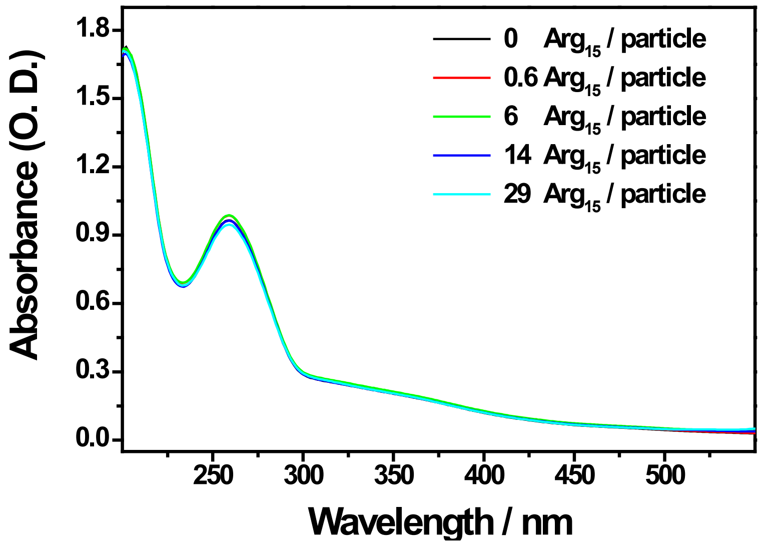

Figure S2. UV-visible spectra of the supernatants after complexation ("step by step") of Li28 and Arg_{15} with iron oxide nanoparticles for the different amounts of Arg_{15}.

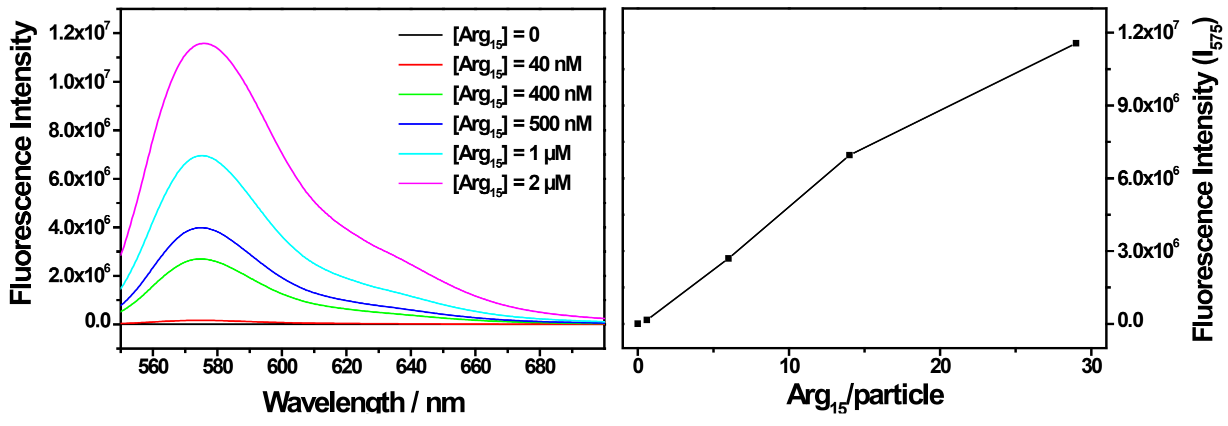

Figure S3. Fluorescence spectra of the TAMRA-labeled Arg_{15} and calibration curve.

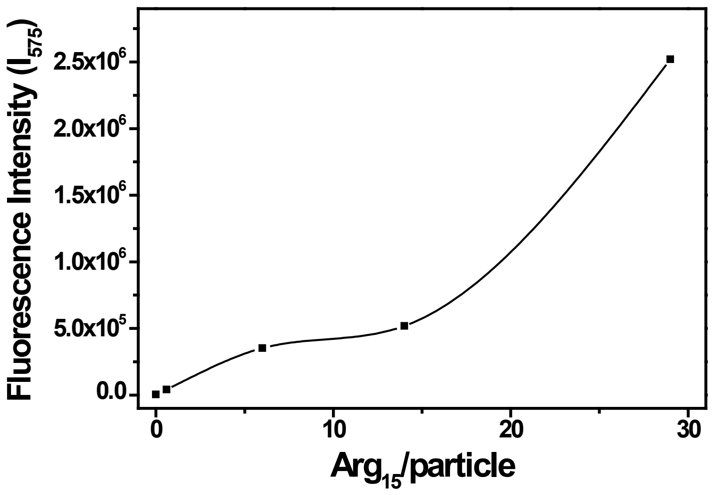

Figure S4. Fluorescence spectra of the supernatants after complexation ("step by step") of Li28 and Arg_{15} with iron oxide nanoparticles for the different amounts of Arg_{15}.

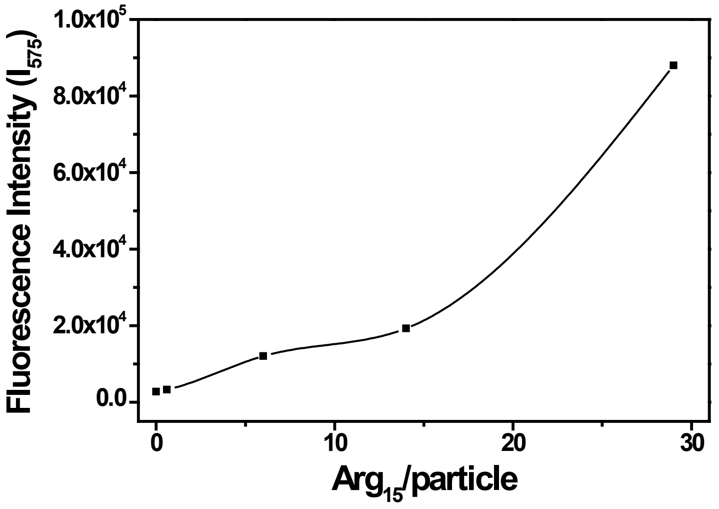

Figure S5. Fluorescence spectra of the complexes (complexation "step by step") of Li28 and Arg_{15} with iron oxide nanoparticles for the different amounts of Arg_{15}.

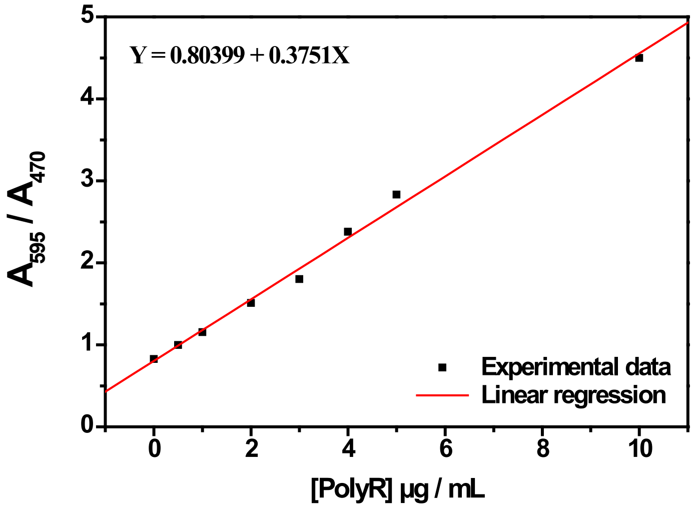

Figure S6. Calibration curve of the Bradford PolyR assay.

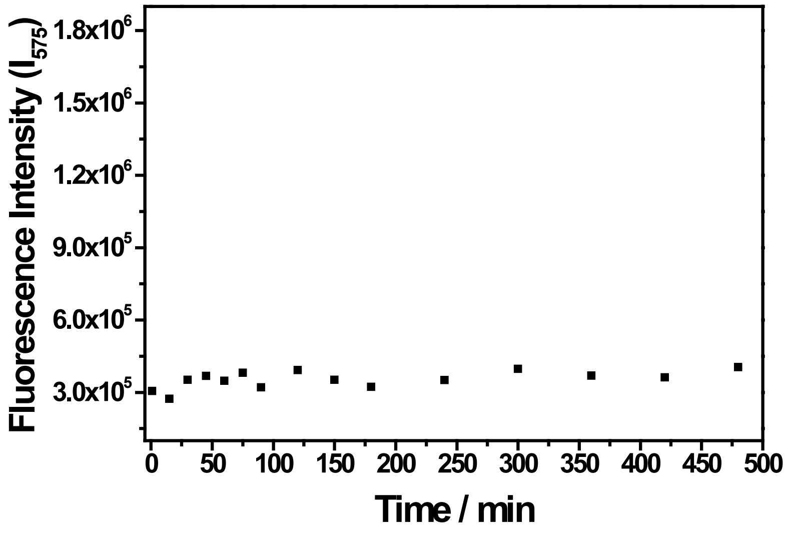

Figure S7. Fluorescence intensity of the nanocomplex in the presence of 10% serum.

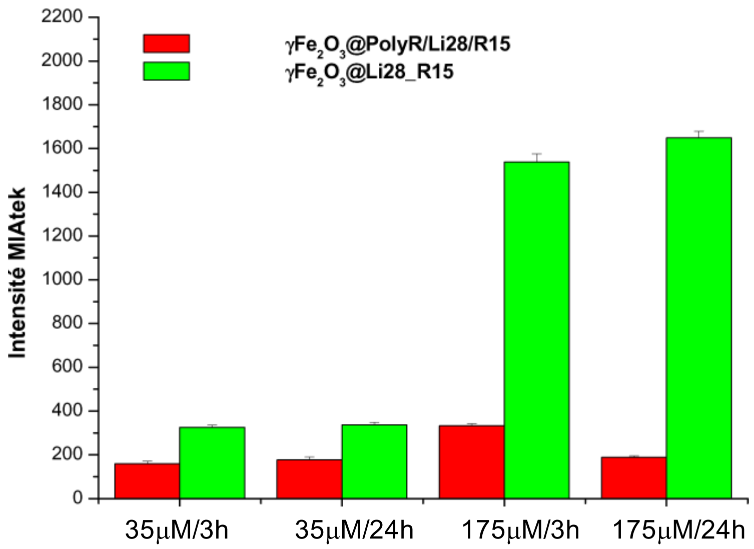

Figure S8. Intracellular internalisation of NPs was quantified with the MIAtek kit, 3 h after incubation with the NPs, and after a subsequent 24-h wash-out period.

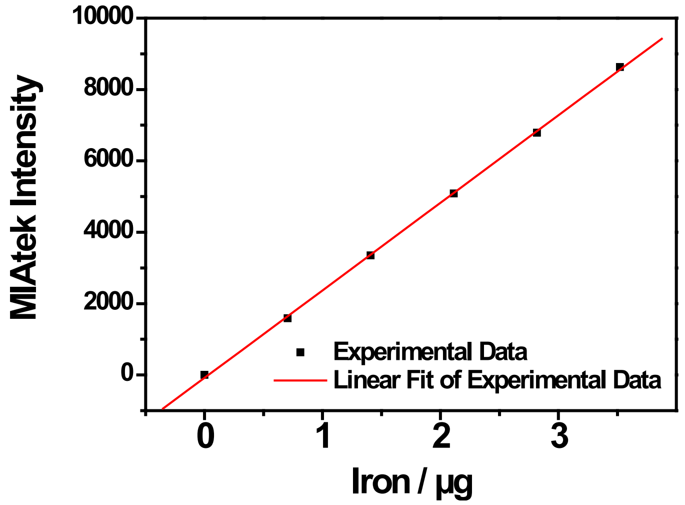

Figure S9. Calibration curve used for the MIAtek quantification.

Table S1. Percentage yield of complexation of Li28 with iron oxide nanoparticles for the different values of *R*.

**Values**	***R***						
	**8.75**	**17.5**	**35**	**52.5**	**70**	**87.5**	**175**
**Yield %**	98.3	98.9	98	98.1	95.9	96.7	-
**Li28/particle**	8.6	17.3	34.3	51.2	67.1	84.6	-

Table S2. Percentage yield of complexation of Arg_{15} (complexation "step by step").

**Values**	**(Arg_{15}/Particle)**				
	**0**	**0.6**	**6**	**14**	**29**
**Yield %**	-	74	87	92	78
**Arg_{15}/particle**	-	0.4	5	13	23

© 2015 by the authors; licensee MDPI, Basel, Switzerland. This article is an open access article distributed under the terms and conditions of the Creative Commons Attribution license (http://creativecommons.org/licenses/by/4.0/).
